# Effect of vasopressin V2-receptor antagonist tolvaptan on syndrome of inappropriate antidiuresis (SIAD) after transsphenoidal pituitary surgery: recovery of measured osmolality

**DOI:** 10.1016/j.heliyon.2022.e10966

**Published:** 2022-10-05

**Authors:** Masahiko Tosaka, Rei Yamaguchi, Yutaro Itabashi, Naoto Mukada, Haruka Tsuneoka, Kentaro Takahashi, Shunsuke Nakamura, Takahiko Nakazawa, Yuhei Yoshimoto

**Affiliations:** Department of Neurosurgery, Gunma University Graduate School of Medicine, Maebashi, Gunma, Japan

**Keywords:** Tolvaptan, Vasopressin V2-receptor antagonist, Hyponatremia, Pituitary, Surgery

## Abstract

**Background:**

Delayed hyponatremia after pituitary surgery can be treated with the V2-receptor antagonist, oral tolvaptan. We investigated the pharmacological effect of oral tolvaptan against SIAD in patients with hyponatremia after pituitary surgery.

**Methods:**

Thirty-nine patients with pituitary adenoma treated by endoscopic transsphenoidal surgery developed SIAD according to the major guidelines, and 7 patients (17.9%) were treated with tolvaptan. Tolvaptan was administrated orally half a tablet (3.75 mg) once in the first two cases, and half a tablet twice in the other five cases. Serum osmolality, urinary osmolality, urinary sodium concentration, urinary volume, and serum sodium and potassium concentration were evaluated before administration, and after the last oral administration of tolvaptan. Serum osmolality and urine osmolality were physically measured.

**Results:**

Serum sodium concentration was significantly increased from 132.1 ± 4.0 to 143.0 ± 2.9 mmol/L (mean ± standard deviation, n = 7, P < 0.001). Serum osmolality was significantly increased from 266.3 ± 7.7 to 289.6 ± 6.7 mOsm/kg (n = 7, P < 0.001). Urine osmolality was significantly reduced from 607.1 ± 240.4 to 262.7 ± 115.6 mOsm/kg (n = 7, P = 0.01). Urinary sodium concentration was significantly decreased from 121.3 ± 48.4 to 36.9 ± 35.0 mOsm/kg (n = 7, P = 0.001). Urine output (24-hour including the first administration) was significantly increased from 1384.2 ± 550.7 to 3291.3 ± 1710.9 mL/day (n = 6, P = 0.026).

**Conclusions:**

Oral tolvaptan administration corrects SIAD after pituitary surgery. Hyponatremia after pituitary surgery was confirmed to be due to SIAD.

## 1 Introduction

Hyponatremia tends to occur about 1 week after surgery for pituitary adenomas. Delayed hyponatremia occurs at a relatively high rate of 3.6%–19.8% of all cases, is transient [1], and causes headache, nausea, vomiting, general fatigue, and other symptoms. Severe hyponatremia may cause disturbance of consciousness and convulsions. Hyponatremia is considered to result from the occurrence of a syndrome of inappropriate antidiuretic hormone (SIADH), but the cause is not completely understood [2, 3, 4, 5]. Postoperative severe hyponatremia can lead to extremely serious complications such as central pontine myelinolysis without rigorous treatment [6]. However, the circulating levels of arginine vasopressin are often not elevated or too unstable to evaluate during hypotonic hyponatremia, such as SIADH.

In general, patients with SIADH do not necessarily have elevated circulating levels of antidiuretic hormone. SIAD was proposed as the correct term for this condition in 2005 [7, 8]. After that, the new term SIAD has become gradually more common [9, 10, 11, 12]. SIAD was proposed as a more accurate description also for this condition after pituitary surgery [13].

Vasopressin-receptor antagonists (vaptans) were found to have effects on SIAD in the SALT study [14]. Conivaptan, the intravenous V1A/V2 -receptor antagonist has been used for the treatment of euvolemic hyponatremia in hospitalized patients, as well as delayed hyponatremia after pituitary surgery, and showed a therapeutic effect on hyponatremia [15]. Tolvaptan, the selective oral V2-receptor antagonist was approved in the U.S. in 2009 for the treatment of clinically significant hypervolemic and euvolemic hyponatremia, including patients with heart failure and SIAD, and in the European Union (EU) for the treatment of hyponatremia secondary to SIAD in adults. It was also approved in Japan in 2020. The tolvaptan is now known to improve hyponatremia after pituitary surgery [16, 17], but any recovery effect on serum osmolality has not yet been demonstrated. Serum sodium concentration is the most important factor in determining serum osmolality, but is not identical.

Here we report the pharmacological effect of oral tolvaptan, V2-receptor antagonist, in patients with SIAD after pituitary surgery.

## 2 Methods

### 2.1 Patients

Thirty-nine patients with pituitary adenoma underwent endoscopic transsphenoidal surgery at Gunma University Hospital from July 2020 to June 2021. Twenty-six cases were non-functioning pituitary adenomas, 10 were growth hormone-producing adenomas, 2 were adrenocorticotropic hormone-producing adenomas, and 1 was thyroid-stimulating hormone-producing adenoma. SIAD was defined according to the current guidelines as follows: (1) plasma osmolality <275 mOsm/kg; (2) inappropriate urine concentration (urinary osmolality >100 mOsm/kg); (3) urine sodium >30 mmol/L; (4) clinical euvolemia; (5) and exclusion of hypothyroidism or secondary adrenal failure [11]. Water intoxication due to the use of antidiuretic hormones (ADHs) was strictly excluded. Three patients with hypothyroidism and decreased serum-free T4 were also excluded. Finally, SIAD was diagnosed in 7 patients (17.9%) and treated with tolvaptan. Three cases were non-functional pituitary adenomas, 3 were growth hormone-producing adenomas, and 1 was prolactin-producing adenoma. Biochemical testing used general test methods, and in-hospital standard values were used. Serum osmolality and urine osmolality were physically measured by the freezing point depression method (measured value) [18]. This study was reviewed and approved by the institutional review board of Gunma University Graduate School of Medicine (HS2021-254). The institutional review boards approved an opt-out method of informed consent.

### 2.2 Treatment for postoperative hyponatremia

Postoperative glucocorticoid replacement was routinely performed. On the day of surgery, hydrocortisone sodium succinate 100 mg was administered and the dose gradually decreased. At 1 week after surgery, when delayed hyponatremia is common, 10–20 mg/day of hydrocortisone was administered orally, so hyponatremia due to adrenal insufficiency is unlikely to occur. Postoperatively, blood sodium levels were examined daily or every other day, and daily total urine output was plotted. Clinical symptoms and decrease in serum sodium concentration were judged to indicate hyponatremia, and water intake restriction was immediately enforced. The rules for tolvaptan administration in Japan only allow use if there is no improvement after water intake restriction. Total water intake was limited to 900–1200 mL for about 24 h. Blood and urine spot sampling during the next morning examined the following simultaneously: (1) Serum osmolality, (2) urinary osmolality, (3) urinary sodium concentration, (4) serum electrolyte (sodium, potassium, chloride) and serum blood urea nitrogen (BUN) levels. Additionally, serum cortisol, adrenocorticotropic hormone (ACTH), free T4 were examined. ADH level was measured in some patients. The normal reference ranges for laboratory parameters in our institute were as follows. Serum osmolality: 275–290 mOsm/L, urinary osmolality: 50–1300 mOsm/L, urinary sodium concentration: not determined (mmol/L), serum sodium: 137–145 mmol/L, potassium chloride: 100–107 mmol/L, BUN: 8–20 g/dL, serum cortisol: 3.0–19.6 μg/dL, ACTH: 7.2–63.3 pg/ml, free T4: 0.75–1.42 ng/dL, and ADH: ≤2.8 pg/ml. Diagnosis of SIAD was based on the diagnostic criteria [11], then tolvaptan (oral distintegration type; OD) 3.75 mg/day (half of a tablet, evening) was administered. All cases were tested in the morning of the day after the start of the tolvaptan. Tolvaptan was administered only half a tablet once (evening) in the first two cases, and twice (evening, and the next morning) in the other five cases. Therefore, the blood and spot urine tests were performed in the first 2 cases approximately 12 h after the last dose, and in the other 5 cases 1–3 h after the last dose. Twenty-four hours urine output from morning to next morning was recorded every day after surgery. The 24-hour urine output the day before the administration was determined as the baseline. The 24-hour urine volume including the first oral administration of tolvaptan was determined as the urine output after oral tolvaptan in this study.

## 3 Results

Clinical and laboratory findings of pre- and post-administration of tolvaptan are summarized in Table 1. Two patients are included who had no symptoms but low sodium level was observed by routine blood sampling. All other patients complained of headache. A patient complained of general fatigue.

**Table 1:** Effect of tolvaptan on syndrome of inappropriate antidiuresis after transsphenoidal pituitary surgery.

Case No.			1	2	3	4	5	6	7
Age/sex			66/M	44/M	77/F	74/F	48/F	63/F	77/F
Type of adenoma			GHPA	GHPA	GHPA	NFPA	PRL	NFPA	NFPA
	Symptoms		mild headache	None	mild headache, general fatigue	none	headache	mild headache	mild headache
Before oral tolvaptan	Post op. day		5	6	10	7	8	5	8
	Daily urine output	mL	-	1270	1300	1730	2210	1230	565
	Na+	mmol/L	131	133	124	132	134	135	136
	K+	mmol/L	4.3	4.2	4.2	3.5	3.9	3.1	2.9
	Serum osmolality	mOsm/kg	265	273	251	272	270	263	270
	Urine osmolality	mOsm/kg	692	969	873	425	352	441	498
	Urine Na+	mmol/L	147	201	88	73	71	153	116
	BUN	mg/dL	20	14	16	11	10	9	11
	ACTH	pg/mL	17.4	40.6	22.8	3.7	15.4	24.7	7.5
	Cortisol	μg/dL	23.3	6.2	4.9	31.2	19.9	10.1	24.2
	fT4	ng/dL	0.92	1.76	1.41	1.47	1.12	1.09	1.35
	ADH	pg/mL	0.6	-	1.6	0.7	0.8	-	-
Oral tolvaptan	3.75 mg per day		1	1	2	2	2	2	2
After oral tolvaptan	Daily urine output	mL	1430	5415	3430	1950	5270	2148	1535
	Na+	mmol/L	142	143	140	140	147	142	147
	K+	mmol/L	4.4	3.8	4.6	4.4	3.8	3.1	3.4
	Serum osmolality	mOsm/kg	287	290	281	286	299	286	298
	Urine osmolality	mOsm/kg	246	409	143	290	95	386	270
	Urine Na+	mmol/L	25	58	16	12	20	109	18
	BUN	mg/dL	16	13	12	13	14	10	15

ACTH, adrenocorticotropic hormone; BUN, serum blood urea nitrogen; GHPA, growth hormone producing pituitary adenoma; NFPA, non-functioning pituitary adenoma; PRL, prolactin-producing pituitary adenoma.

**Figure 1:**
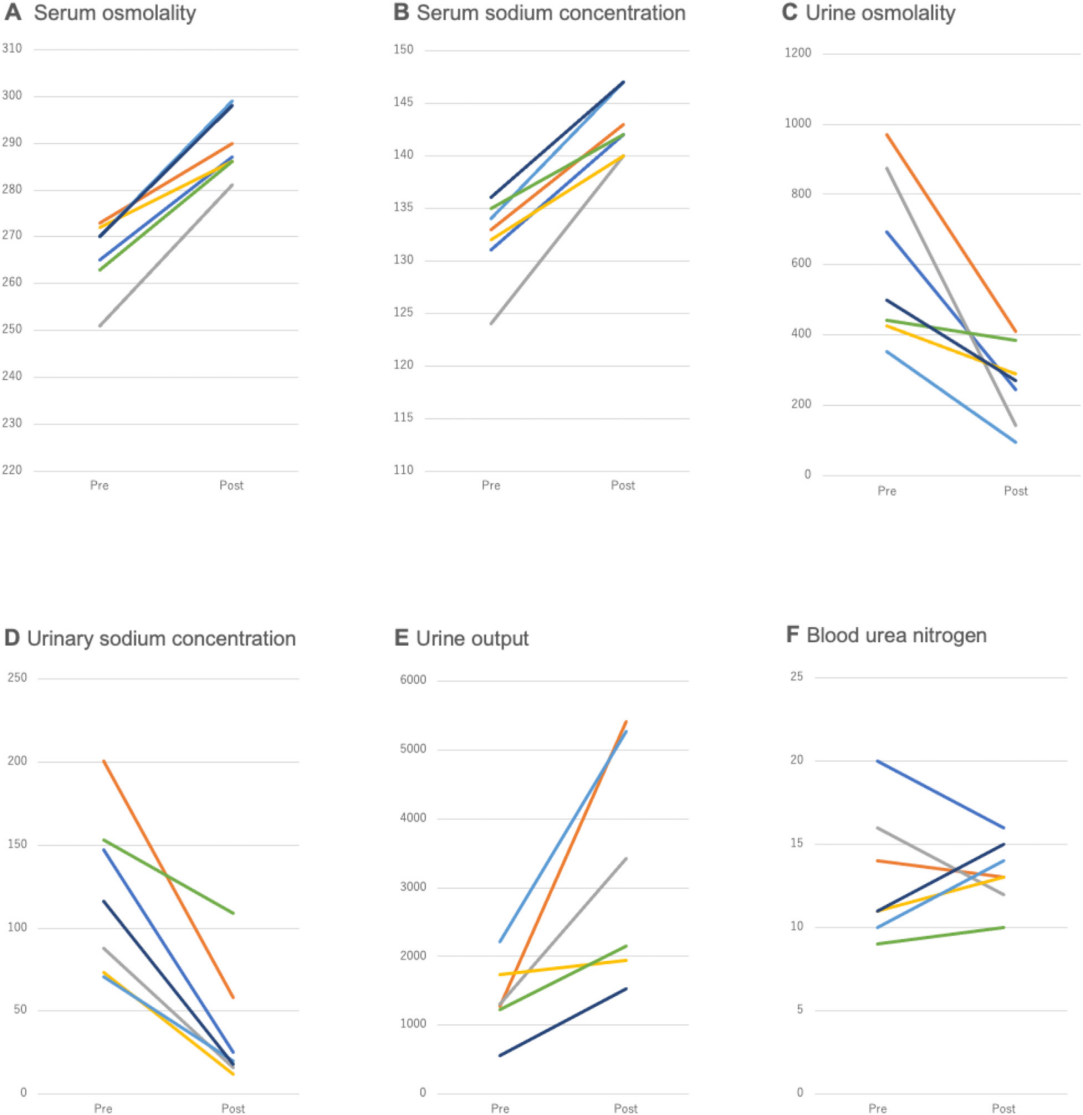
(A) Serum osmolality (mOsm/kg) showed significant increase (n = 7, P < 0.001) ter tolvaptan administration. (B) Serum sodium concentration (mmol/L) was significantly increased (n = 7, P < 0.001). (C) Urine osmolality (mOsm/kg) was significantly reduced (n = 7, P = 0.01). (D) Urinary sodium concentration (mmol/L) showed significant decrease (n = 7, P = 0.001). (E) Urine output (mL) was significantly increased (n = 6, P = 0.026). (F) Blood urea nitrogen (BUN) (mg/dL) showed no significant difference before and after administration. The same color line indicates the same patient.

Serum sodium concentration, osmolality, urine osmolality, urinary sodium concentration, urine output, and BUN concentration before and after tolvaptan administration are shown in Figure 1(A, B, C, D, E, F). Serum sodium concentration was significantly increased after tolvaptan administration from 132.1 ± 4.0 to 143.0 ± 2.9 mmol/L (mean ± standard deviation, n = 7, P < 0.001). Serum osmolality also showed a significant increase from 266.3 ± 7.7 to 289.6 ± 6.7 mOsm/kg (n = 7, P < 0.001). Urine osmolality was significantly reduced from 607.1 ± 240.4 to 262.7 ± 115.6 mOsm/kg (n = 7, P = 0.01). Urinary sodium concentration showed a significant decrease from 121.3 ± 48.4 to 36.9 ± 35.0 mOsm/kg (n = 7, P = 0.001). Urine output was significantly increased from 1384.2 ± 550.7 to 3291.3 ± 1710.9 mL/day (n = 6, P = 0.026). Serum potassium concentration showed no significant difference from 3.7 ± 0.6 to 3.9 ± 0.6 mmol/L (n = 7, P = 0.27). Serum BUN showed no significant difference from 13.0 ± 3.9 to 13.3 ± 2.0 mmol/L (n = 7, P = 0.83). Adrenocorticotropic hormone value of 18.9 ± 12.2 pg/mL, cortisol value of 17.1 ± 10.1 pg/mL (with administration of 10–20 mg/day of hydrocortisone), and free T4 level of 1.3 ± 0.28 ng/dL showed no decrease. ADH was measured in 4 patients at 0.6, 1.6, 0.7, and 0.8 pg/mL, which tended to be rather low. The effects of tolvaptan on serum sodium concentration and osmolality showed similar slopes. The effects of tolvaptan for lowering urinary osmotic pressure and urinary sodium concentration were also confirmed. The urinary sodium concentration decreased and the urine volume increased, with some variations (Figure 1(A, B, C, D, E, F)). In all cases, sodium levels normalized within 2 days.

## 4 Discussion

Tolvaptan administration was used for the treatment of SIAD after pituitary surgery, which confirmed that hyponatremia, osmolality, urine osmolality, and urinary sodium concentration, all subsequently recovered. Tolvaptan administration is effective to correct SIAD after pituitary surgery. Since tolvaptan improved measured serum osmolality, SIAD was confirmed as the mechanism of hyponatremia after pituitary surgery. We conclude that tolvaptan can correct hyponatremia that occurs after pituitary surgery, which meets the standard criteria for SIAD even if not severe. Low tolvaptan doses of 3.75 mg/day seemed to be effective to correct hyponatremia after pituitary surgery in Japanese patients.

Serum sodium concentration and serum osmolality are not perfectly proportional [19]. Recently, the effect of tolvaptan on postoperative hyponatremia after pituitary surgery were reported only using sodium concentration as an index [13, 16]. Indirli et al. used tolvaptan for SIAD after pituitary surgery. However, the effect of tolvaptan was only confirmed by an increase in serum sodium concentration, which may not be sufficient to conclude that any corrective effect on SIAD [13]. For the first time, this case series confirmed that tolvaptan reverses SIAD after pituitary surgery using measured osmolality [18].

In recent years, SIAD has been considered to cause delayed hyponatremia after pituitary surgery, and the consensus is that treatment with water restriction is appropriate [4, 5]. However, the cause of delayed hyponatremia after pituitary surgery is not fully understood. For example, cerebral salt wasting syndrome can also cause low sodium levels [20]. Postoperative adrenal insufficiency could also cause hyponatremia [21, 22]. In patients with SIAD, such as our present patients (n = 4), ADH levels are often not sufficiently elevated even with hyponatremia. Our study confirmed that administration of V2-receptor antagonist could change all SIAD indicators, including osmolality as well as sodium concentration in SIAD after pituitary surgery. Conversely, this pharmacological evidence may suggest that SIAD is the cause of delayed hyponatremia after pituitary surgery.

Postoperative patients with pituitary adenoma are often followed up by hospitalization for about 1–2 weeks after surgery in Japan. Sodium concentration is routinely evaluated one week after surgery. Therefore, SIAD may be diagnosed earlier than elsewhere. The definition of hyponatremia may be debatable. We ordered water restriction and osmotic pressure measurement when the patient complained of headache (majority) about 1 week after surgery, even if the serum sodium was 136 mmol/L, but lower than before surgery [23, 24]. If SIAD was diagnosed, tolvaptan was administered (Cases 6 and 7). Even in these patients, the V2-receptor antagonist was clearly effective. SIAD could be diagnosed even if the sodium concentration did not necessarily decrease below 135 mmol/L. Hyponatremia is not included in classical diagnostic features of SIADH [25], or the standard criteria of SIAD [11]. The present study demonstrated that the hyponatremia that occurs after pituitary surgery is dilution hyponatremia due to the retention of free water, which may be better named “SIAD after pituitary surgery” than “hyponatremia after pituitary surgery.” This study found that sodium movement after tolvaptan administration was more similar to osmolality movement than any other factors. Therefore, only the sodium concentration is generally safe to discuss in the setting of SIAD. However, dilution hyponatremia should be understood in SIAD after pituitary surgery. Water restriction, or administration of V2-receptor antagonists such as tolvaptan, is an essential treatment strategy. The number of cases may not be sufficient for definitive conclusions. This retrospective single arm study investigated the effects of oral tolvaptan. In the future, serum osmolality should be used as an index for comparative trial of simple water restriction and drug treatment, and larger prospective studies.

## Declarations

### Author contribution statement

Tosaka M: Conceived and designed the experiments; Analyzed and interpreted the data; Wrote the paper.

Yamaguchi R: Conceived and designed the experiments; Analyzed and interpreted the data.

Yoshimoto Y: Conceived and designed the experiments; Wrote the paper.

Itabashi Y: Performed the experiments; Analyzed and interpreted the data; Contributed reagents, materials, analysis tools or data.

Mukada N: Performed the experiments; Contributed reagents, materials, analysis tools or data.

Tsuneoka H: Performed the experiments; Analyzed and interpreted the data.

Takahashi K, Nakamura S and Nakazawa T: Performed the experiments.

### Funding statement

This research did not receive any specific grant from funding agencies in the public, commercial, or not-for-profit sectors.

### Data availability statement

Data included in article/supp. material/referenced in article.

### Declaration of interest’s statement

The authors declare the following conflict of interests: The first author is cooperating in a post-marketing surveillance of vasopressin V2-receptor antagonist Tolvaptan (Samska OD tablets®️, Otsuka Parmaceutical Co., Ltd).

### Additional information

No additional information is available for this paper.
